# Modulation of *APOE* and *SORL1* genes on hippocampal functional connectivity in healthy young adults

**DOI:** 10.1007/s00429-017-1377-3

**Published:** 2017-02-22

**Authors:** Junlin Shen, Wen Qin, Qiang Xu, Lixue Xu, Jiayuan Xu, Peng Zhang, Huaigui Liu, Bing Liu, Tianzi Jiang, Chunshui Yu

**Affiliations:** 1 Department of Radiology and Tianjin Key Laboratory of Functional Imaging, Tianjin Medical University General Hospital, No. 154, Anshan Road, Heping District, Tianjin, 300052 China; 2 Brainnetome Center, Institute of Automation, Chinese Academy of Sciences, Beijing, China

**Keywords:** *APOE*, *SORL1*, Hippocampus, Functional connectivity, fMRI, SNPs

## Abstract

Apolipoprotein E (*APOE*) and sortilin-related receptor (*SORL1*) genes act on the same metabolic pathway and have been associated with Alzheimer’s disease (AD) characterized by hippocampal impairment. Although the effects of *APOE* on hippocampal resting-state functional connectivity (rsFC) have been reported, the main effects of *SORL1* and *SORL1* × *APOE* interactions on hippocampal rsFC in healthy subjects remain largely unknown. Here, we systematically investigated the main effects of *SORL1 rs2070045*, and *APOE*, and their interaction effects on hippocampal rsFC in healthy young adults. The main effect of *APOE* showed that risk ε4 carriers had decreased positive hippocampal rsFC with the precuneus/posterior cingulate cortex and subgenual anterior cingulate cortex, and increased positive hippocampal rsFC with the sensorimotor cortex compared with non-ε4 carriers. The main effect of *SORL1* showed that risk G-allele carriers had decreased positive rsFC between the hippocampus and middle temporal gyrus compared with TT carriers. No significant additive interaction was observed. Instead, significant *SORL1* × *APOE* non-additive interaction was found in negative rsFC between the hippocampus and inferior frontal gyrus. Compared with subjects with TT genotype, *SORL1* G-allele carriers had a stronger negative rsFC in *APOE* ε4 carriers, but a weaker negative rsFC in *APOE* non-ε4 carriers. These findings suggest that *SORL1* and *APOE* genes modulate different hippocampal rsFCs and have a complex interaction. The *SORL1-* and *APOE-*dependent hippocampal connectivity changes may at least partly account for their association with AD.

**Electronic supplementary material:**

The online version of this article (doi:10.1007/s00429-017-1377-3) contains supplementary material, which is available to authorized users.

## Introduction

As a common polygenic disorder, Alzheimer’s disease (AD) is clinically characterized by progressive deterioration of memory and other cognitive abilities, and is pathologically characterized by formation of senile plaques and neurofibrillary tangles (Bird 2008). The ε4-allele of apolipoprotein E gene (*APOE* ε4) is a chief genetic risk factor for late-onset AD (Verghese et al. 2011), but it is neither necessary nor sufficient for AD (Bertram and Tanzi 2008; Slooter et al. 1998). Genome-wide association studies (GWAS) have associated AD with several other genetic variations, including sortilin-related receptor (*SORL1*) gene (Rogaeva et al. 2007). Several previous studies have reported *SORL1* × *APOE* interactions on the risk for AD (Cellini et al. 2009; Kimura et al. 2009) and on amyloid protein β (Aβ) concentrations in cerebrospinal fluid (CSF) in AD patients (Alexopoulos et al. 2011a, b). This interaction may be mediated by the bind of SORL1 to APOE ligand, which induces endocytosis of APOE-containing lipoproteins (Taira et al. 2001).

Understanding genetic effects on brain imaging phenotypes may help to identify potential pathways from gene to disease. Because hippocampal atrophy is the most prominent feature of AD pathology (Hill et al. 2014), many imaging genetics studies have explored associations between AD-related genetic variants and hippocampal atrophy. For example, *APOE* ε4 carriers have shown greater hippocampal atrophy than non-carriers in AD patients, cognitively normal elderly, and healthy young adults (Alexopoulos et al. 2011a, b; den Heijer et al. 2002; O’Dwyer et al. 2012; Pievani et al. 2011). *SORL1* risk allele has also been related to hippocampal atrophy in AD patients (Cuenco et al. 2008) and in healthy young subjects (Bralten et al. 2011). The functional disconnection between the hippocampus and neocortical regions is another feature in AD impairments (Wang et al. 2006). *APOE* genotypes have been associated with hippocampal resting-state functional connectivity (rsFC) in healthy adults (Fleisher et al. 2009; Heise et al. 2014; Sheline et al. 2010). However, it remains unclear whether and how *SORL1* genetic variation modulates hippocampal rsFC. Both *APOE* and *SORL1* act on the amyloid precursor protein (APP) pathway (Bohm et al. 2015) and affect the hippocampus (Louwersheimer et al. 2015; Pievani et al. 2011), suggesting a potential interaction between *APOE* and *SORL1*.

In the present study, we collected data from 287 healthy young subjects and used an imaging genetic method to investigate the main effects of *SORL1* and *APOE*, and their interactions on hippocampal rsFC, which may provide new insight on the role of *SORL1* and *APOE* in AD pathology.

## Materials and methods

### Participants

A total of 287 healthy, young, right-handed subjects (134 males and 153 females; mean age: 22.7 ± 2.4 years, ranging from 18 to 29 years) were selected from 323 subjects who participated in this study. Fifteen subjects were excluded because of poor image quality. Two subjects were excluded because of genotyping failure for *APOE* status, and three subjects with ε2ε4 genotype were also excluded because of the opposite effects of the two alleles. Thirteen subjects were excluded because of genotyping failure for *SORL1*. Memory function was assessed by the Chinese Revised Wechsler Memory Scale (RC-WMS). Memory quotient was used to assess global memory ability, and the visual reproduction subscale was used to assess episodic memory. Three subjects were further excluded due to lack of memory data. All participants were carefully screened to ensure that they had no history of psychiatric or neurological illness, and had no contraindications for MRI examinations. To purify the sample, only Chinese Han subjects were recruited. The study protocol was approved by the Medical Research Ethics Committee of Tianjin Medical University, and written informed consent was obtained from each participant.

### Genotyping

We extracted genomic DNA from 3000 µl of the whole blood using the EZgeneTM Blood gDNA Miniprep Kit (BiomigaInc, San Diego, CA, USA). The standard protocols were used to genotypes *SORL1 rs2070045* and *APOE*. Detailed methods are described in Supplementary Materials. On the basis of *APOE* ε4 status (Verghese et al. 2011), subjects were divided into ε4 carriers and non-carriers. Because most studies have indicated that G-allele of *SORL1 rs2070045* is risk allele for AD (Reitz et al. 2011), subjects were further subdivided into G-allele carriers and TT homozygotes.

### Image acquisition

MR images were acquired using a Signa HDx 3.0 T MR scanner (General Electric, Milwaukee, WI, USA). Tight but comfortable foam padding was used to minimize head movement, and earplugs were used to reduce scanner noise. Resting-state fMRI data were obtained using Gradient-Echo Single-Shot Echo-Planar Imaging sequence (GRE-SS-EPI) with the following imaging parameters: repetition time (TR)/echo time (TE) = 2000/30 ms; field of view (FOV) = 240 mm × 240 mm; matrix = 64 × 64; flip angle (FA) = 90°; slice thickness = 4 mm; no gap; 40 interleaved transversal slices; and 180 volumes. During fMRI scans, all subjects were instructed to keep their eyes closed to stay as still as possible, to think of nothing in particular, and to not fall asleep. Sagittal 3D T1-weighted images were acquired by a brain-volume sequence (TR/TE = 8.1/3.1 ms; inversion time = 450 ms; FA = 13°; FOV = 256 mm × 256 mm; matrix = 256 × 256; slice thickness = 1 mm, no gap; and 176 slices).

### Data preprocessing

Before data preprocessing, we carefully examined the imaging quality of each subject and did not find unacceptable artifact in any subject. Resting-state fMRI data were preprocessed using the Statistical Parametric Mapping (SPM8, http://www.fil.ion.ucl.ac.uk/spm) and Data Processing Assistant for Resting-State fMRI (DPARSF) (Chao-Gan and Yu-Feng 2010). The first ten volumes of each functional time series were discarded to allow signal to reach equilibrium and the participants to adapt to scanning noise. The remaining 170 volumes were corrected for acquisition time delay between slices and were realigned to the first volume. Head movement parameters were estimated, and each volume was realigned to the mean map to correct for geometrical displacements using a six-parameter rigid-body transformation. Fifteen subjects were excluded from further analysis, because their maximum displacement in any of the three orthogonal directions was more than 2 mm or a maximum rotation was greater than 2.0°. We also calculated framewise displacement, which indexes volume-to-volume changes in head position. These changes were obtained from derivatives of the rigid-body realignment estimates that were used to realign fMRI data (Power et al. 2012, 2013). Subsequently, individual structural images were co-registered to the mean functional image with a linear transformation. The transformed structural images were then segmented into gray matter, white matter, and cerebrospinal fluid using a unified segmentation algorithm (Ashburner and Friston 2005). The motion-corrected functional volumes were spatially normalized to the Montreal Neurological Institute (MNI) space and re-sampled to 3 × 3 × 3 mm^3^ voxels using the same transformation parameters. The normalized fMRI data were smoothed with a full width at half-maximum (FWHM) of 6 mm. Several sources of spurious variances, including estimated motion parameters, linear drift, and average fMRI signals in the whole brain, ventricle, and white matter regions, were removed from the data using linear regression. Finally, temporal band-pass filtering (0.01–0.08 Hz) was performed on time series of each voxel to reduce the effects of low-frequency drift and high-frequency noises (Liu et al. 2013).

### rsFC analysis

**Fig. 1:**
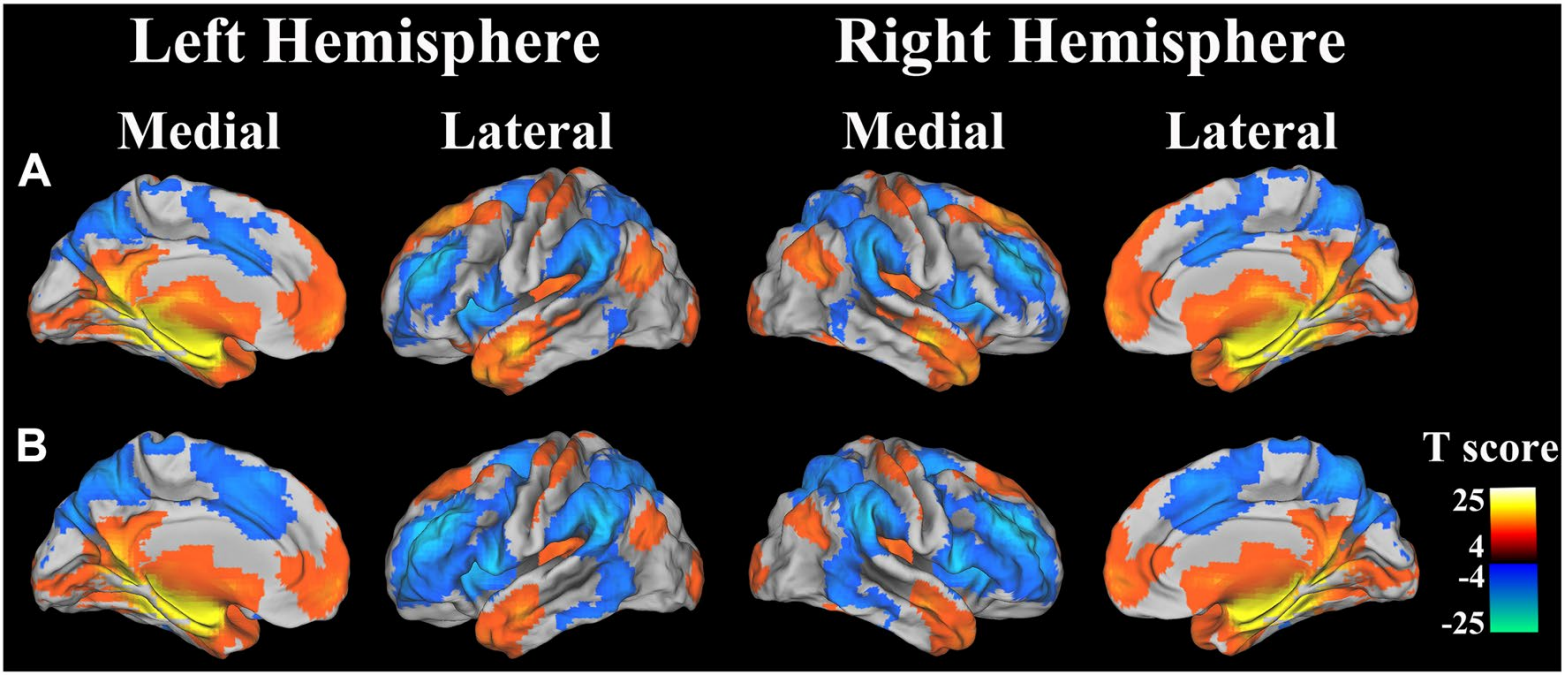
rsFC patterns of hippocampus. a Left hippocampus; and b right hippocampus. Positive (*warm color*) and negative (*Cold color*) correlations are projected to structural images. *Color scales* present *T* value of one-sample *t* test

The left and right hippocampal seed regions were extracted from the Harvard-Oxford Subcortical Structure Atlas using a probability threshold of 50%. For each individual, Pearson correlation coefficient between the mean time series of each seed region and that of each voxel throughout the whole brain was calculated (Liu et al. 2015). The resulting correlation coefficients were transformed into *z* values using Fisher’s *z* transformation. Then, individuals’ *z* values were entered into a random effect one-sample *t* test in a voxelwise manner to identify brain regions that showed significant correlations with the seed region. Multiple comparisons were corrected for familywise error (FWE) with a threshold of *P* < 0.05. Thus, the whole brain rsFC maps of left and right hippocampus were created (Fig. 1).

### Gray matter volume (GMV) analysis

GMV was calculated by SPM8 (http://www.fil.ion.ucl.ac.uk/spm/software/spm8). Detailed methods are described in Supplementary Materials. We defined brain regions with significant rsFC differences with the hippocampus as regions of interest (ROIs). The GMVs of these ROIs were extracted and compared between genotypes (*P* < 0.05, uncorrected).

### Statistical analysis

Hardy–Weinberg equilibrium was tested using the Chi-square goodness-of-fit test. Statistical analyses for demographic, head motion, and psychological data were performed using the Statistical Package for the Social Sciences version 18.0 (SPSS) for Windows (*P* < 0.05). Comparisons between genetic subgroups were performed using a one-way analysis of variance (ANOVA) for continuous variables (age, years of education, and framewise displacement), and a Chi-square test for categorical variable (sex). A two-way analysis of covariance (ANCOVA) was used to assess the main effects of *SORL1* and *APOE* and their interactions on memory quotient and visual reproduction score while controlling for age, gender, and years of education.

For the combined gene analyses of *APOE* and *SORL1*, we tested for both additive interaction effects and non-additive interaction effects. Two-way ANCOVA was used to assess the main effects of *SORL1* and *APOE* and non-additive interactions between *SORL1* and *APOE* on hippocampal rsFC controlling for age, gender, and years of education. Although default settings in SPM and SPSS calculate Type III sums of squares to deal with unbalanced data, a nonparametric model may be another reasonable option. Permutation is such a nonparametric test that requires few assumptions about the data and is a reliable method to cope with unbalanced data (Winkler et al. 2014). Thus, we performed permutation tests using the Randomise tool of FMRIB Software Library (FSL) (http://fsl.fmrib.ox.ac.uk/fsl/fslwiki/randomise/theory) (Mcfarquhar 2016) to further validate our results. Gender, age, and years of education were included as covariates. Multiple comparisons were corrected using AlphaSim method (*P* < 0.05), which is realized by Monte Carlo simulation. The underlying principle is that true regions of activation will tend to occur over contiguous voxels, but noise is unlikely to form clusters of activated voxels. Thus, the presence of clustering can be used as one criterion to distinguish between signal and noise. The power of the statistical test is largely enhanced by combining probability and cluster thresholding. The program generates an estimate of the overall significance level achieved for various combinations of probability and cluster thresholds. Several parameters should be input to run the program, including the probability threshold at a single voxel level, number of simulation, smoothing kernel, connection fashion of nearby voxels, mask for analysis, and resolution of voxels. The parameters of this study were as follows: single voxel *P* = 0.01, 5000 simulations, and edge connection; with a positive or negative rsFC mask and a resolution of 3 × 3 × 3 mm^3^. After that, the program will generate a series of cluster thresholds corresponding to the selected corrected probability thresholds. For a corrected threshold of *P* < 0.05, the cluster threshold was 20 voxels for the positive rsFC analysis and 25 voxels for the negative rsFC analysis.

To test for additive interaction effects, we created three gene–gene cohorts based on the number of risk alleles in *APOE* and *SORL1*. Participants with *APOE* non-ε4 and *SORL1* TT (0 risk allele) were classified into “1 lowest-risk” cohort; participants with either carriers of *APOE* ε4 and *SORL1* TT or carriers of *APOE* non-ε4 and *SORL1* G-allele (1 risk allele) were classified into “2 middle-risk” cohort; and participants with *APOE* ε4 and *SORL1* G-allele (2 risk alleles) were classified into “3 highest-risk” cohort. Additive effects were tested by a voxelwise linear regression with the degrees of risk as independent factor (1, 2, or 3) and hippocampal rsFC as dependent factor, controlling for age, gender, and years of education. Multiple comparisons were corrected using the same statistical threshold as the previous voxel-based analyses.

Partial correlation analysis was conducted to assess correlations (*P* < 0.05) between memory scores (memory quotient and visual reproduction score) and hippocampal connectivity while controlling for the effects of age, gender, and years of education.

## Results

### Demographic and genetic characteristics

A total of 287 healthy young Chinese Han subjects with high-quality imaging data and *SORL1* and *APOE* genotypic information were finally included in the present study. These subjects were divided into four groups according to genotypes. The demographic data of these groups are shown in Table 1. Both *SORL1* and *APOE* genotypic distributions were in Hardy–Weinberg equilibrium (*P* = 0.20 for *SORL1* and *P* = 0.85 for *APOE*) for whom genotyped successfully. One-way ANOVA revealed that there were no significant differences between the four genotypic groups in age (*P* = 0.25), years of education (*P* = 0.97), and framewise displacement (*P* = 0.80). Chi-square tests did not reveal any significant differences in gender distribution of the four groups (*P* = 0.71). However, we found that *APOE* ε4 carriers had significantly reduced memory quotient (*F* = 6.71, *P* = 0.01) than non-ε4 carriers for the main effect of *APOE* (Fig. S1). In addition, we also found a significant *SORL1-APOE* interaction (*F* = 5.50, *P* = 0.02) on the memory quotient. The *post hoc* analysis showed that subjects with risk *APOE* ε4 allele showed significantly reduced memory quotient (*t* = −2.32, *P* = 0.03) than non-ε4 carriers only in *SORL1* TT carriers (Fig. S2). However, we did not find a significant main effect of *SORL1* on memory quotient and any significant main and interaction effects of the two SNPs on visual reproduction score (*P* > 0.05).

**Table 1:** Genetic, demographic, and psychological characteristics

Combined genotypes	G-allele/ε4 (*N* = 30)	G-allele/non-ε4 (*N* = 193)	TT/ε4 (*N* = 11)	TT/non-ε4 (*N* = 53)
*SORL1* genotype counts GG/GT/TT	9/21/0	86/107/0	0/0/11	0/0/53
*APOE* genotype counts ε4/ε3/ε2	30/0/0	0/30/163	11/0/0	0/45/8
Age (years)	22.2 (2.2)	22.9 (2.4)	22.2 (3.1)	22.3 (2.3)
Gender (M/F)	15/15	93/100	5/6	21/32
Years of education	15.7 (1.9)	15.6 (2.2)	15.7 (1.7)	15.5 (2.3)
Memory quotient	114.7 (8.1)	114.6 (9.3)	108.3 (11.1)	116.8 (10.9)
Visual reproduction score	12.1 (1.2)	12.0 (1.6)	12.3 (1.0)	11.9 (1.6)

The data are shown as means (SD). G-allele = GG + GT; non-ε4 = ε3 + ε2; F, female; M, male

### The main effect of SORL1

**Fig. 2:**
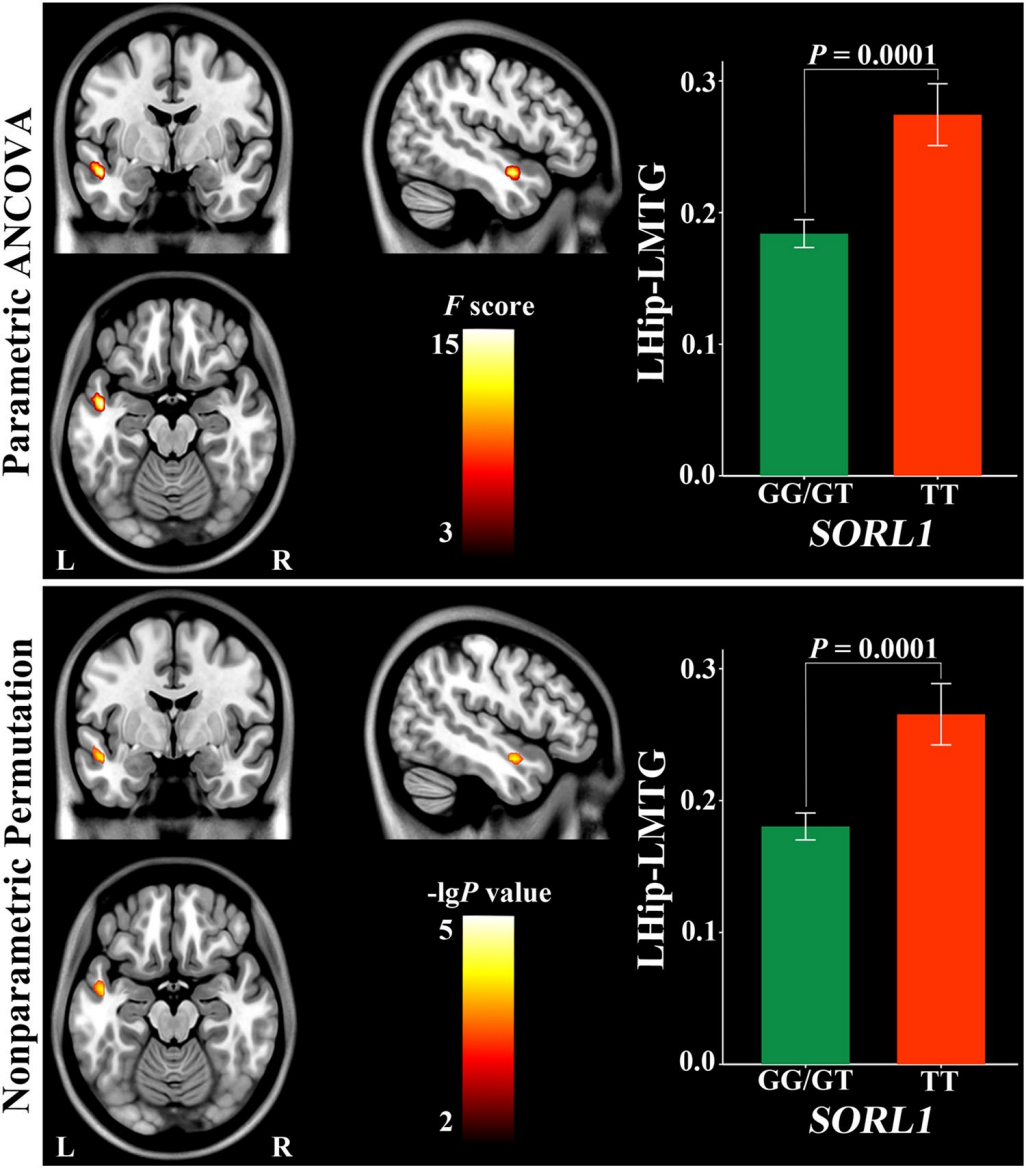
Main effect of *SORL1* on left hippocampal positive connectivity. *Hip* hippocampus, *L* left, *MTG* middle temporal gyrus, *R* right

Using parametric ANCOVA, the main effect of *SORL1* was found in positive rsFC between the left hippocampus and middle temporal gyrus (MTG) (Fig. 2) (Table 2). G-allele (risk genotype) carriers had a weaker positive rsFC than TT (protective genotype) carriers (*P* = 0.0001). None of negative hippocampal rsFC exhibited a significant main effect of *SORL1*.

**Table 2:** Main and interaction effects of *SORL1* and *APOE* on hippocampal connectivity

	Connectivity (sign)	BA	Parametric ANCOVA					Nonparametric permutation				
			Coordinates (MNI)			Cluster size (voxels)	*F* value	Coordinates (MNI)			Cluster size (voxels)	*P* value
			x	y	z			x	y	z		
*SORL1*	LHip and LMTG (positive)	21	−48	−3	−18	26	17.00	−51	−3	−15	34	0.0001
*APOE*	RHip and PCC (positive)	29	−6	−42	18	28	10.53	−6	−42	18	33	0.0009
	RHip and Pcu (positive)	23	12	−60	27	34	12.51	9	−63	27	41	0.0005
	RHip and RSMC (positive)	4	54	−15	51	130	15.42	48	−15	54	144	0.0001
	RHip and LSMC (positive)	4	−42	−30	66	37	11.40	−42	−30	66	64	0.0003
	LHip and Pcu (positive)	23	–	–	–	–	–	12	−57	27	58	0.0014
	LHip and sACC (positive)	25	3	3	−9	45	10.18	3	3	−9	71	0.0006
*SORL1* × *APOE*	RHip and LIFG (negative)	45	−54	33	18	35	11.11	−51	39	0	40	0.0009

*BA* Brodmann area, *Hip* hippocampus, *IFG* inferior frontal gyrus, *L* left, *MTG* middle temporal gyrus, *PCC* posterior cingulate cortex, *Pcu* precuneus, *R* right, *sACC* subgenual anterior cingulate cortex, *SMC* sensorimotor cortex

Using nonparametric permutation, the main effect of *SORL1* was found in the same positive rsFC between the left hippocampus and MTG **(**Fig. 2) (Table 2). G-allele (risk genotype) carriers also had a weaker positive rsFC than TT (protective genotype) carriers (*P* = 0.0001).

### The main effect of APOE

**Fig. 3:**
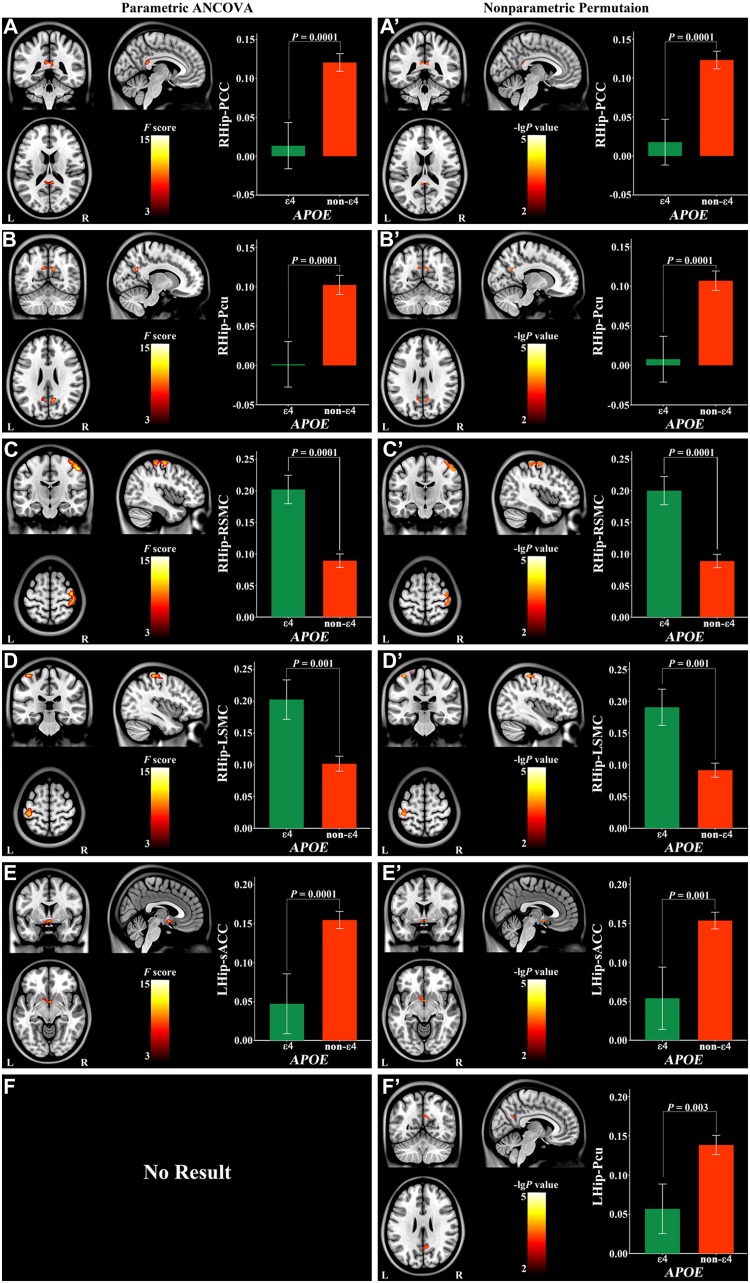
Main effect of *APOE* on hippocampal positive connectivity. *Hip* hippocampus, *L* left, *PCC* posterior cingulate cortex, *Pcu* precuneus, *R* right, *sACC* subgenual anterior cingulate cortex, *SMC* sensorimotor cortex

Using parametric ANCOVA, the main effect of *APOE* was found in positive right hippocampal rsFC with the posterior cingulate cortex (PCC) (Fig. 3a), precuneus (Pcu) (Fig. 3b) and bilateral sensorimotor cortices (SMC) (Fig. 3c, d), and left hippocampal rsFC with the subgenual anterior cingulate cortex (sACC) (Fig. 3e; Table 2). The ε4 (risk genotype) carriers had a weaker positive hippocampal rsFC than non-ε4 (protective genotype) carriers (*P* = 0.0001 for PCC; *P* = 0.0001 for Pcu; *P* = 0.0001 for sACC) and a stronger positive rsFC in bilateral SMC (*P* = 0.0001 for the right; *P* = 0.001 for the left). None of the negative rsFC of the hippocampus exhibited a significant main effect of *APOE*.

Using nonparametric permutation, the main effect of *APOE* was found in positive rsFC of the right hippocampus with the PCC (Fig. 3a′), Pcu (Fig. 3b′), and bilateral SMC (Fig. 3c′, d′; Table 2). In addition, the main effect of *APOE* also existed in positive rsFC of the left hippocampus with the sACC and Pcu (Fig. 3e′, f′; Table 2). The ε4 (risk genotype) carriers had a weaker positive hippocampal rsFC than non-ε4 (protective genotype) carriers with the PCC (*P* = 0.0001), Pcu (*P* = 0.0001 for right hippocampus; *P* = 0.003 for left hippocampus) and sACC (*P* = 0.001), and a stronger positive hippocampal rsFC with the bilateral SMC (*P* = 0.0001 for the right; *P* = 0.001 for the left).

### Non-additive interactions of SORL1 and APOE

**Fig. 4:**
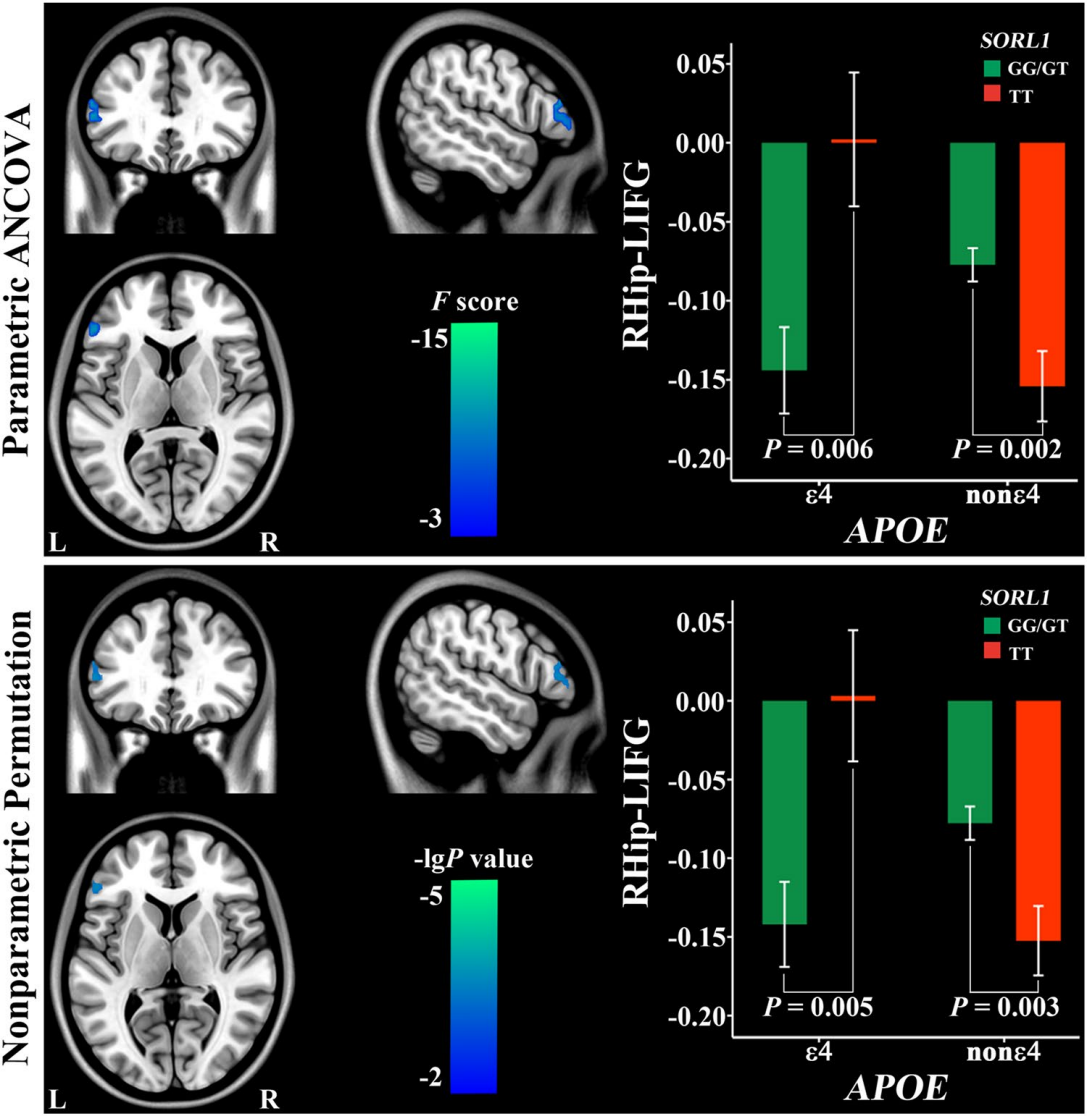
Non-additive interaction of *SORL1* and *APOE* on the right hippocampal negative connectivity. *Hip* hippocampus, *IFG* inferior frontal gyrus, *L* left, *R* right

Using parametric ANCOVA, the non-additive interaction effect of *SORL1* and *APOE* was found only in negative rsFC between the right hippocampus and the left inferior frontal gyrus (IFG) (Fig. 4; Table 2). In risk *APOE* ε4 carriers, subjects with risk *SORL1* G-allele had a stronger negative rsFC than those with protective TT (*P* = 0.006). In *APOE* non-ε4 carriers, in contrast, negative rsFC was weaker in subjects with risk *SORL1* G-allele than in those with TT (*P* = 0.002).

Using nonparametric permutation, the non-additive interaction effect of *SORL1 and APOE* was found in the same negative rsFC between the right hippocampus and the IFG (Fig. 4; Table 2). In risk *APOE* ε4 carriers, subjects with risk *SORL1* G-allele had a stronger negative rsFC than those with protective TT (*P* = 0.005). In APOE non-ε4 carriers, in contrast, negative rsFC was weaker in subjects with risk SORL1 G-allele than in those with TT (*P* = 0.003).

### Comparison of rsFC between double and one risk-allele carriers

To clarify the relationship between increased and decreased hippocampal rsFC in risk-allele carriers, we extracted hippocampal rsFC values with significant intergroup differences from each risk-allele carrier, and then compared rsFC differences between double and one risk-allele carriers using general linear model while controlling for the effects of age, sex, and educational years. For hippocampal rsFC with significant main effect of *APOE*, compared with one risk-allele carriers (*APOE* ε4-allele or *SORL1* G-allele), double carriers (*APOE* ε4-allele + *SORL1* G-allele) showed weaker right hippocampal rsFC with the PCC (*P* = 0.001) (Fig. S3) and the Pcu (*P* = 0.004) (Fig. S4). For hippocampal rsFC with significant main effect of *SORL1*, compared with one risk-allele carriers, double carriers showed a trend towards rsFC reduction between the left hippocampus and the left MTG (*P* = 0.07) (Fig. S5). For hippocampal rsFC with significant interactive effect of *SORL1* and *APOE*, compared with one risk-allele carriers, double carriers showed stronger right hippocampal rsFC with the left IFG (*P* = 0.01) (Fig. S6).

We also compared rsFC differences of the IFG with the MTG, PCC, and PCu between double- and single risk-allele carriers. The IFG-MTG (*SORL1*-related region) (Fig. S7), IFG-PCC (*APOE*-related region) (Fig. S8), and IFG-Pcu (*APOE*-related region) (Fig. S9) rsFCs did not differ between double and single risk-allele carriers.

### Additive interactions of SORL1 and APOE

We did not find any significant additive interaction effects of the degrees of risk on hippocampal rsFCs under the same statistical threshold (*P* < 0.05, AlphaSim correction).

### Correlations between hippocampal connectivity and memory scores

For all hippocampal rsFCs with a significant main or interaction effect of *SORL1* and *APOE*, we calculated correlations between these hippocampal rsFCs and memory scores. Only the left hippocampal rsFC with the Pcu was significantly associated with memory quotients (*P* = 0.001) and showed a trend towards significant correlation with visual reproduction scores (*P* = 0.07). Other hippocampal rsFCs did not show any significant correlations (*P* < 0.05).

### GMV differences

To determine whether rsFC of brain regions with significant genotypic difference were associated with GMV differences across genotypes, we compared GMVs of these clusters and bilateral hippocampi. However, neither significant main effects nor *APOE* × *SORL1* interactions were found in GMVs of these ROIs (*P* > 0.05).

## Discussion

In the present study, we systematically investigated the main effects and interactions of *SORL1* and *APOE* genetic variations on hippocampal rsFC in healthy young adults. The main effect analyses showed that carriers with *APOE* risk ε4 had reduced or increased hippocampal rsFC, while *SORL1* risk G-allele had reduced hippocampal rsFC compared with carriers with a protective genotype. Moreover, we showed a significant non-additive interaction of *SORL1* and *APOE*, but failed to show any significant additive effects.

APOE, a transporter of cholesterol and lipids, plays a critical role in lipid homeostasis and neuronal repair in the brain (Mahley et al. 2006). As a chief known genetic risk factor (Verghese et al. 2011), *APOE* ε4 increases the risk for late-onset AD by binding to β-amyloid protein and accelerating deposition of amyloid (Mahley et al. 2006). However, *APOE* ε4 alone is neither necessary nor sufficient for AD (Bertram and Tanzi 2008; Slooter et al. 1998). GWAS has identified several genomic regions that are associated with AD susceptibility, including *SORL1* gene (Rogaeva et al. 2007). *SORL1* genetic variants cause reduced expression of SORL1 in the brain in AD (Scherzer et al. 2004). Lack of SORL1 switches the amyloid precursor protein (APP) away from retromer recycling pathway, and instead directs APP into β-secretase cleavage pathway, increasing APPsβ production and then into γ-secretase cleavage pathway to generate Aβ peptide (Rogaeva et al. 2007). Most studies support *SORL1 rs2070045* G-allele as the risk genetic factor for late-onset AD, including the evidence from Chinese Han population (Reitz et al. 2011). SORL1 binds multiple ligands including APOE and plays a role in endocytosis of APOE-containing lipoproteins (Taira et al. 2001). Interactions between *SORL1* and *APOE* might interfere with the formation of APOE-Aβ complex, and this process may foster Aβ deposition in the brain by increasing unbound Aβ species that leading to pathogenesis of AD (Ikeuchi et al. 2010).

Both hippocampus and PCC/Pcu are brain regions that show the earliest changes of AD pathology (Braak and Braak 1991; Buckner et al. 2005). The normal anatomical and functional connections between these two regions are critically important for successful memory formation (Miller et al. 2008). Disruption of this connection has been proposed as an early imaging biomarker for AD and a primary factor in episodic memory impairment associated with early AD (Zhou et al. 2008). Using a seed-based rsFC analysis, *APOE* ε4 carriers show decreased rsFC between hippocampus and PCC/Pcu than non-ε4 carriers in normal elderly subjects (Heise et al. 2014), even in normal subjects without Aβ deposits (Sheline et al. 2010). Using the same analysis method, we extended this finding into healthy young adults, suggesting that *APOE* ε4-allele modulates this rsFC decades prior to the typical age at onset of AD. In contrast to the finding of ε4-allele-related connectivity reduction, independent component analysis (ICA) has revealed increased hippocampal connectivity with the default mode network (DMN) in *APOE* ε4 carriers in healthy young adults (Filippini et al. 2009) and in middle-aged and elderly healthy subjects (Westlye et al. 2011). Different analytical approaches may account for the discrepancy. Seed-based rsFC measures temporal correlation of BOLD signal fluctuation between the seed and each voxel of the brain. In contrast, ICA measures temporal synchronization of BOLD signal fluctuation between a network (such as the DMN) and each voxel of this network. In hippocampal rsFC analysis, each significant cluster has strong connectivity with the hippocampus. However, the hippocampus is not the core component of the DMN derived from ICA.

The sensorimotor cortex has been considered to be relatively spared of AD pathology (Braak and Braak 1991; Suvà et al. 1999). However, there is increasing evidence for sensorimotor dysfunction early in the disease (Albers et al. 2015). In addition, fMRI studies have revealed that decreased or rewired sensorimotor network connectivity in AD, even at an early stage (Agosta et al. 2010; Brier et al. 2012; Damoiseaux et al. 2012; Dipasquale et al. 2015; Wang et al. 2015a, b). Evidence from behavioral studies in elderly individuals indicates that *APOE* ε4 carriers have an enhanced vulnerability for impaired motor function (Buchman et al. 2009; Carmelli et al. 2000; Melzer et al. 2005). A resting-state network study reveals that the sensorimotor network exhibits decreased connectivity in healthy elders with *APOE* ε4 (Wang et al. 2015a, b). On the contrary, in healthy young subjects, *APOE* ε4 carriers exhibit increased functional connectivity in the sensorimotor network (Filippini et al. 2009). Our study showed that *APOE* ε4 carriers showed increased rsFC between the hippocampus and sensorimotor cortex than non-ε4 carriers in healthy young subjects. The opposite main effects of *APOE* on hippocampal rsFC with the sensorimotor cortex (increased in ε4 carriers) and PCC/Pcu (decreased in ε4 carriers) may reflect intrinsically anti-correlated relationship between task-negative DMN and task-positive sensorimotor networks (Fox et al. 2005).

The sACC has shown perfusion decrease from entorhinal to limbic stages during the process of AD pathology (Bradley et al. 2002). A significant GMV and metabolic decrease also occurs in the sACC at an early stage of AD (Fouquet et al. 2009; Frisoni et al. 2009). Moreover, metabolic reduction in the sACC has been correlated with that in the hippocampus in patients with mild cognitive impairment (Fouquet et al. 2009), which has been linked to anatomical disconnection between the two regions (Villain et al. 2010). Resting-state fMRI data have shown that the sACC is functionally connected with the hippocampus (Yu et al. 2011). Therefore, decreased hippocampal rsFC with the sACC in young healthy *APOE* ε4 carriers may be an indicator for the AD risk.

Pathological study has revealed that MTG is one of the neocortical sites that are early affected in AD (Braak and Braak 1991). Longitudinal structural analysis has shown that MTG atrophy occurs shortly after hippocampal atrophy and is secondary to hippocampal changes during the course of AD (Li et al. 2011). Resting-state fMRI studies have revealed that rsFC between hippocampus and MTG is significantly decreased in AD patients (Allen et al. 2007; Wang et al. 2006). Our study revealed that young healthy *SORL1* G carriers (risk allele) exhibited decreased hippocampal rsFC with MTG. This finding suggests that connectivity impairment between these two regions already starts in young adults at genetic risk for AD, which may predispose *SORL1* G-allele carriers to be susceptible for AD after several decades.

The IFG is involved in cognitive control of memory, permitting memory to be accessed strategically (Badre and Wagner 2007). The IFG and hippocampus are interconnected via polysynaptic pathways (Barredo et al. 2015) and are co-activated during memory tasks (Dove et al. 2006). In this study, we found a non-additive *APOE*-*SORL1* interaction on the hippocampus-IFG connectivity. In non-ε4 carriers, *SORL1* risk-allele carriers had weaker hippocampus-IFG connectivity than non-carriers, which can be explained by the main effect of the *SORL1*. However, in ε4 carriers, *SORL1* risk-allele carriers had stronger hippocampus-IFG negative connectivity than non-carriers. Because double risk-allele carriers showed reduced hippocampal connectivity with the MTG, PCC, and Pcu than single risk-allele carriers, the increased hippocampus-IFG negative connectivity in double carriers may compensate for the connectivity impairment of the hippocampus. Alternatively, the increased hippocampus-IFG negative connectivity in double carriers may reflect activity changes in these two regions (hippocampus and IFG) in the opposite direction, because AD patients show increased activation in the IFG and decreased activation in the MTL during both encoding and retrieving processes (Schwindt and Black 2009). Further studies are needed to clarify the biological relevance of the increased hippocampus-IFG negative connectivity in double carriers.

We also investigated effects of *APOE* and *SORL1* on GMVs of the hippocampus and brain regions whose rsFC with the hippocampus exhibiting a significant genetic modulation effect. However, we did not find any significant main effects and interactions, suggesting that effects of *APOE* and *SORL1* on hippocampal rsFCs are not likely to be the result of GMV changes in these regions. The lack of a modulation effect of *APOE* on hippocampal volume in young healthy adults is consistent with several previous studies (Khan et al. 2014; Mondadori et al. 2007; Richter-Schmidinger et al. 2011; Sidiropoulos et al. 2011), but inconsistent with others (Alexopoulos et al. 2011a, b; O’Dwyer et al. 2012). Future studies with a large sample sizes are needed to clarify the issue.

Much evidence demonstrates that *APOE* ε4 is associated with memory deficits in healthy elders (Caselli et al. 2009; Wisdom et al. 2011). In healthy young adults, however, inconsistent findings have been reported on the association between *APOE* status and memory. One study shows that *APOE* ε4 is related to better memory score (Mondadori et al. 2007); however, this finding has not been replicated in another study (Bunce et al. 2011). In this study, young *APOE* ε4 carriers had significantly reduced memory quotient and hippocampus-Pcu connectivity than non-ε4 carriers regardless of their SORL1 status. Because hippocampus-Pcu connectivity is critically important for memory formation (Miller et al. 2008), the positive correlation between memory quotients and this connectivity suggests that reduced hippocampus-Pcu connectivity may underlie memory deficit in *APOE* ε4 carriers. Of course, further efforts are warranted to clarify the discrepancy of memory performance in young *APOE* ε4 carriers across studies.

Several limitations should be noted when interpreting our findings. Only Chinese Han subjects were included in this study. Investigations on other ethnic populations may provide information on whether our finding is a generalized effect across ethnic populations. Brain regions with significant genotypic difference were not found to be correlated with visual reproduction test in our sample. RC-WMS may not be the most sensitive scale for assessing memory function, more powerful neuropsychological tests are needed in future study. Only healthy young subjects were included in this study. Investigation on the effects of *SORL1* and *APOE* gene on brain structural and functional changes in other developmental stages and in patients with memory deficit or AD may provide us a more complete understanding of the effects of *SORL1* and *APOE* genetic variations. Because global signal has been thought to reflect non-neuronal noise, global signal regression (GSR) has been used as a standard step during processing of resting-state fMRI data. However, GSR may induce spurious negative correlations. Since there is no clear consensus on what such correlations mean in terms of “connectivity”, the *SORL1*-*APOE* interaction effect on the hippocampus-IFG negative connectivity should be carefully interpreted.

In conclusion, the current results showed altered hippocampal rsFC in carriers with risk *APOE* ε4 or *SORL1* G-allele, which may predispose these risk-allele carriers to be susceptible for AD after several decades. We also showed a non-additive interaction of *SORL1* and *APOE*, suggesting the complexity of the effects of AD-related genetic variations.

## Electronic supplementary material

Below is the link to the electronic supplementary material.

Supplementary material 1 (DOC 8232 KB)

## References

[CR1] Agosta F, Rocca MA, Pagani E, Absinta M, Magnani G, Marcone A, Falautano M, Comi G, Gorno-Tempini ML, Filippi M (2010). Sensorimotor network rewiring in mild cognitive impairment and Alzheimer’s disease. Hum Brain Mapp.

[CR2] Albers MW, Gilmore GC, Kaye J, Murphy C, Wingfield A, Bennett DA, Boxer AL, Buchman AS, Cruickshanks KJ, Devanand DP, Duffy CJ, Gall CM, Gates GA, Granholm AC, Hensch T, Holtzer R, Hyman BT, Lin FR, McKee AC, Morris JC, Petersen RC, Silbert LC, Struble RG, Trojanowski JQ, Verghese J, Wilson DA, Xu S, Zhang LI (2015). At the interface of sensory and motor dysfunctions and Alzheimer’s disease. Alzheimers Dement.

[CR3] Alexopoulos P, Guo LH, Kratzer M, Westerteicher C, Kurz A, Perneczky R (2011). Impact of SORL1 single nucleotide polymorphisms on Alzheimer’s disease cerebrospinal fluid markers. Dement Geriatr Cogn Disord.

[CR4] Alexopoulos P, Richter-Schmidinger T, Horn M, Maus S, Reichel M, Sidiropoulos C, Rhein C, Lewczuk P, Doerfler A, Kornhuber J (2011). Hippocampal volume differences between healthy young apolipoprotein E epsilon2 and epsilon4 carriers. J Alzheimers Dis.

[CR5] Allen G, Barnard H, McColl R, Hester AL, Fields JA, Weiner MF, Ringe WK, Lipton AM, Brooker M, McDonald E, Rubin CD, Cullum CM (2007). Reduced hippocampal functional connectivity in Alzheimer disease. Arch Neurol.

[CR6] Ashburner J, Friston KJ (2005). Unified segmentation. Neuroimage.

[CR7] Badre D, Wagner AD (2007). Left ventrolateral prefrontal cortex and the cognitive control of memory. Neuropsychologia.

[CR8] Barredo J, Oztekin I, Badre D (2015). Ventral fronto-temporal pathway supporting cognitive control of episodic memory retrieval. Cereb Cortex.

[CR9] Bertram L, Tanzi RE (2008). Thirty years of Alzheimer’s disease genetics: the implications of systematic meta-analyses. Nat Rev Neurosci.

[CR10] Bird TD (2008). Genetic aspects of Alzheimer disease. Genet Med.

[CR11] Bohm C, Chen F, Sevalle J, Qamar S, Dodd R, Li Y, Schmitt-Ulms G, Fraser PE, St George-Hyslop PH (2015). Current and future implications of basic and translational research on amyloid-beta peptide production and removal pathways. Mol Cell Neurosci.

[CR12] Braak H, Braak E (1991). Neuropathological stageing of Alzheimer-related changes. Acta Neuropathol.

[CR13] Bradley KM, O’Sullivan VT, Soper ND, Nagy Z, King EM, Smith AD, Shepstone BJ (2002). Cerebral perfusion SPET correlated with Braak pathological stage in Alzheimer’s disease. Brain.

[CR14] Bralten J, Arias-Vasquez A, Makkinje R, Veltman JA, Brunner HG, Fernandez G, Rijpkema M, Franke B (2011). Association of the Alzheimer’s gene SORL1 with hippocampal volume in young, healthy adults. Am J Psychiatry.

[CR15] Brier MR, Thomas JB, Snyder AZ, Benzinger TL, Zhang D, Raichle ME, Holtzman DM, Morris JC, Ances BM (2012). Loss of intranetwork and internetwork resting state functional connections with Alzheimer’s disease progression. J Neurosci.

[CR16] Buchman AS, Boyle PA, Wilson RS, Beck TL, Kelly JF, Bennett DA (2009). Apolipoprotein E e4 allele is associated with more rapid motor decline in older persons. Alzheimer Dis Assoc Disord.

[CR17] Buckner RL, Snyder AZ, Shannon BJ, LaRossa G, Sachs R, Fotenos AF, Sheline YI, Klunk WE, Mathis CA, Morris JC, Mintun MA (2005). Molecular, structural, and functional characterization of Alzheimer’s disease: evidence for a relationship between default activity, amyloid, and memory. J Neurosci.

[CR18] Bunce D, Anstey KJ, Burns R, Christensen H, Easteal S (2011). Does possession of apolipoprotein E ɛ4 benefit cognitive function in healthy young adults?. Neuropsychologia.

[CR19] Carmelli D, DeCarli C, Swan GE, Kelly-Hayes M, Wolf PA, Reed T, Guralnik JM (2000). The joint effect of apolipoprotein E epsilon4 and MRI findings on lower-extremity function and decline in cognitive function. J Gerontol A Biol Sci Med Sci.

[CR20] Caselli RJ, Dueck AC, Osborne D, Sabbagh MN, Connor DJ, Ahern GL, Baxter LC, Rapcsak SZ, Shi J, Woodruff BK, Locke DE, Snyder CH, Alexander GE, Rademakers R, Reiman EM (2009). Longitudinal modeling of age-related memory decline and the APOE epsilon4 effect. N Engl J Med.

[CR21] Cellini E, Tedde A, Bagnoli S, Pradella S, Piacentini S, Sorbi S, Nacmias B (2009). Implication of sex and SORL1 variants in italian patients with Alzheimer disease. Arch Neurol.

[CR22] Chao-Gan Y, Yu-Feng Z (2010). DPARSF: A MATLAB toolbox for “Pipeline” data analysis of resting-state fMRI. Front Syst Neurosci.

[CR23] Cuenco KT, Lunetta KL, Baldwin CT, McKee AC, Guo J, Cupples LA, Green RC, St George-Hyslop PH, Chui H, DeCarli C, Farrer LA (2008). Association of distinct variants in SORL1 with cerebrovascular and neurodegenerative changes related to Alzheimer disease. Arch Neurol.

[CR24] Damoiseaux JS, Prater KE, Miller BL, Greicius MD (2012). Functional connectivity tracks clinical deterioration in Alzheimer’s disease. Neurobiol Aging.

[CR25] den Heijer T, Oudkerk M, Launer LJ, van Duijn CM, Hofman A, Breteler MM (2002). Hippocampal, amygdalar, and global brain atrophy in different apolipoprotein E genotypes. Neurology.

[CR26] Dipasquale O, Griffanti L, Clerici M, Nemni R, Baselli G, Baglio F (2015). High-Dimensional ICA Analysis Detects Within-Network Functional Connectivity Damage of Default-Mode and Sensory-Motor Networks in Alzheimer’s Disease. Front Hum Neurosci.

[CR27] Dove A, Brett M, Cusack R, Owen AM (2006). Dissociable contributions of the mid-ventrolateral frontal cortex and the medial temporal lobe system to human memory. Neuroimage.

[CR28] Filippini N, MacIntosh BJ, Hough MG, Goodwin GM, Frisoni GB, Smith SM, Matthews PM, Beckmann CF, Mackay CE (2009). Distinct patterns of brain activity in young carriers of the APOE-epsilon4 allele. Proc Natl Acad Sci USA.

[CR29] Fleisher AS, Sherzai A, Taylor C, Langbaum JB, Chen K, Buxton RB (2009). Resting-state BOLD networks versus task-associated functional MRI for distinguishing Alzheimer’s disease risk groups. Neuroimage.

[CR30] Fouquet M, Desgranges B, Landeau B, Duchesnay E, Mézenge F, de la Sayette V, Viader F, Baron JC, Eustache F, Chételat G (2009). Longitudinal brain metabolic changes from amnestic mild cognitive impairment to Alzheimer’s disease. Brain.

[CR31] Fox MD, Snyder AZ, Vincent JL, Corbetta M, Van Essen DC, Raichle ME (2005). The human brain is intrinsically organized into dynamic, anticorrelated functional networks. Proc Natl Acad Sci USA.

[CR32] Frisoni GB, Prestia A, Rasser PE, Bonetti M, Thompson PM (2009). In vivo mapping of incremental cortical atrophy from incipient to overt Alzheimer’s disease. J Neurol.

[CR33] Heise V, Filippini N, Trachtenberg AJ, Suri S, Ebmeier KP, Mackay CE (2014). Apolipoprotein E genotype, gender and age modulate connectivity of the hippocampus in healthy adults. Neuroimage.

[CR34] Hill DL, Schwarz AJ, Isaac M, Pani L, Vamvakas S, Hemmings R, Carrillo MC, Yu P, Sun J, Beckett L, Boccardi M, Brewer J, Brumfield M, Cantillon M, Cole PE, Fox N, Frisoni GB, Jack C, Kelleher T, Luo F, Novak G, Maguire P, Meibach R, Patterson P, Bain L, Sampaio C, Raunig D, Soares H, Suhy J, Wang H, Wolz R, Stephenson D (2014). Coalition against major diseases/european medicines agency biomarker qualification of hippocampal volume for enrichment of clinical trials in predementia stages of Alzheimer’s disease. Alzheimers Dement.

[CR35] Ikeuchi T, Hirayama S, Miida T, Fukamachi I, Tokutake T, Ebinuma H, Takubo K, Kaneko H, Kasuga K, Kakita A, Takahashi H, Bujo H, Saito Y, Nishizawa M (2010). Increased levels of soluble LR11 in cerebrospinal fluid of patients with Alzheimer disease. Dement Geriatr Cogn Disord.

[CR36] Khan W, Giampietro V, Ginestet C, Dell’Acqua F, Bouls D, Newhouse S, Dobson R, Banaschewski T, Barker GJ, Bokde AL, Buchel C, Conrod P, Flor H, Frouin V, Garavan H, Gowland P, Heinz A, Ittermann B, Lemaitre H, Nees F, Paus T, Pausova Z, Rietschel M, Smolka MN, Strohle A, Gallinat J, Westman E, Schumann G, Lovestone S, Simmons A (2014). No differences in hippocampal volume between carriers and non-carriers of the ApoE epsilon4 and epsilon2 alleles in young healthy adolescents. J Alzheimers Dis.

[CR37] Kimura R, Yamamoto M, Morihara T, Akatsu H, Kudo T, Kamino K, Takeda M (2009). SORL1 is genetically associated with Alzheimer disease in a Japanese population. Neurosci Lett.

[CR38] Li X, Coyle D, Maguire L, Watson DR, McGinnity TM (2011). Gray matter concentration and effective connectivity changes in Alzheimer’s disease: a longitudinal structural MRI study. Neuroradiology.

[CR01] Liu F, Guo W, Liu L, Long Z, Ma C, Xue Z, Wang Y, Li J, Hu M, Zhang J, Du H, Zeng L, Liu Z, Wooderson SC, Tan C, Zhao J, Chen H (2013) Abnormal amplitude low-frequency oscillations in medication-naive, first-episode patients with major depressive disorder: a resting-state fMRI study. J Affect Disord 146:401–406 10.1016/j.jad.2012.10.001 23116810

[CR02] Liu F, Guo W, Fouche JP, Wang Y, Wang W, Ding J, Zeng L, Qiu C, Gong Q, Zhang W, Chen H (2015) Multivariate classification of social anxiety disorder using whole brain functional connectivity. Brain Struct Funct 220:101–115 10.1007/s00429-013-0641-4 24072164

[CR39] Louwersheimer E, Ramirez A, Cruchaga C, Becker T, Kornhuber J, Peters O, Heilmann S, Wiltfang J, Jessen F, Visser PJ, Scheltens P, Pijnenburg YA, Teunissen CE, Barkhof F, van Swieten JC, Holstege H, Van der Flier WM (2015). The influence of genetic variants in SORL1 gene on the manifestation of Alzheimer’s disease. Neurobiol Aging.

[CR40] Mahley RW, Weisgraber KH, Huang Y (2006). Apolipoprotein E4: a causative factor and therapeutic target in neuropathology, including Alzheimer’s disease. Proc Natl Acad Sci USA.

[CR41] Mcfarquhar M (2016). Testable Hypotheses for Unbalanced Neuroimaging Data. Front Neurosci.

[CR42] Melzer D, Dik MG, van Kamp GJ, Jonker C, Deeg DJ (2005). The apolipoprotein E e4 polymorphism is strongly associated with poor mobility performance test results but not self-reported limitation in older people. J Gerontol A Biol Sci Med Sci.

[CR43] Miller SL, Celone K, DePeau K, Diamond E, Dickerson BC, Rentz D, Pihlajamaki M, Sperling RA (2008). Age-related memory impairment associated with loss of parietal deactivation but preserved hippocampal activation. Proc Natl Acad Sci USA.

[CR44] Mondadori CR, de Quervain DJ, Buchmann A, Mustovic H, Wollmer MA, Schmidt CF, Boesiger P, Hock C, Nitsch RM, Papassotiropoulos A, Henke K (2007). Better memory and neural efficiency in young apolipoprotein E epsilon4 carriers. Cereb Cortex.

[CR45] O’Dwyer L, Lamberton F, Matura S, Tanner C, Scheibe M, Miller J, Rujescu D, Prvulovic D, Hampel H (2012). Reduced hippocampal volume in healthy young ApoE4 carriers: an MRI study. PLoS One.

[CR46] Pievani M, Galluzzi S, Thompson PM, Rasser PE, Bonetti M, Frisoni GB (2011). APOE4 is associated with greater atrophy of the hippocampal formation in Alzheimer’s disease. Neuroimage.

[CR47] Power JD, Barnes KA, Snyder AZ, Schlaggar BL, Petersen SE (2012). Spurious but systematic correlations in functional connectivity MRI networks arise from subject motion. Neuroimage.

[CR48] Power JD, Barnes KA, Snyder AZ, Schlaggar BL, Petersen SE (2013). Steps toward optimizing motion artifact removal in functional connectivity MRI; a reply to Carp. Neuroimage.

[CR49] Reitz C, Cheng R, Rogaeva E, Lee JH, Tokuhiro S, Zou F, Bettens K, Sleegers K, Tan EK, Kimura R, Shibata N, Arai H, Kamboh MI, Prince JA, Maier W, Riemenschneider M, Owen M, Harold D, Hollingworth P, Cellini E, Sorbi S, Nacmias B, Takeda M, Pericak-Vance MA, Haines JL, Younkin S, Williams J, van Broeckhoven C, Farrer LA, St George-Hyslop PH, Mayeux R (2011). Meta-analysis of the association between variants in SORL1 and Alzheimer disease. Arch Neurol.

[CR50] Richter-Schmidinger T, Alexopoulos P, Horn M, Maus S, Reichel M, Rhein C, Lewczuk P, Sidiropoulos C, Kneib T, Perneczky R, Doerfler A, Kornhuber J (2011). Influence of brain-derived neurotrophic-factor and apolipoprotein E genetic variants on hippocampal volume and memory performance in healthy young adults. J Neural Transm (Vienna).

[CR51] Rogaeva E, Meng Y, Lee JH, Gu Y, Kawarai T, Zou F, Katayama T, Baldwin CT, Cheng R, Hasegawa H, Chen F, Shibata N, Lunetta KL, Pardossi-Piquard R, Bohm C, Wakutani Y, Cupples LA, Cuenco KT, Green RC, Pinessi L, Rainero I, Sorbi S, Bruni A, Duara R, Friedland RP, Inzelberg R, Hampe W, Bujo H, Song YQ, Andersen OM, Willnow TE, Graff-Radford N, Petersen RC, Dickson D, Der SD, Fraser PE, Schmitt-Ulms G, Younkin S, Mayeux R, Farrer LA, St George-Hyslop P (2007). The neuronal sortilin-related receptor SORL1 is genetically associated with Alzheimer disease. Nat Genet.

[CR52] Scherzer CR, Offe K, Gearing M, Rees HD, Fang G, Heilman CJ, Schaller C, Bujo H, Levey AI, Lah JJ (2004). Loss of apolipoprotein E receptor LR11 in Alzheimer disease. Arch Neurol.

[CR53] Schwindt GC, Black SE (2009). Functional imaging studies of episodic memory in Alzheimer’s disease: a quantitative meta-analysis. Neuroimage.

[CR54] Sheline YI, Morris JC, Snyder AZ, Price JL, Yan Z, D’Angelo G, Liu C, Dixit S, Benzinger T, Fagan A, Goate A, Mintun MA (2010). APOE4 allele disrupts resting state fMRI connectivity in the absence of amyloid plaques or decreased CSF Aβ42. J Neurosci.

[CR55] Sidiropoulos C, Jafari-Khouzani K, Soltanian-Zadeh H, Mitsias P, Alexopoulos P, Richter-Schmidinger T, Reichel M, Lewczuk P, Doerfler A, Kornhuber J (2011). Influence of brain-derived neurotrophic factor and apolipoprotein E genetic variants on hemispheric and lateral ventricular volume of young healthy adults. Acta Neuropsychiatr.

[CR56] Slooter AJ, Cruts M, Kalmijn S, Hofman A, Breteler MM, Van Broeckhoven C, van Duijn CM (1998). Risk estimates of dementia by apolipoprotein E genotypes from a population-based incidence study: the Rotterdam Study. Arch Neurol.

[CR57] Suvà D, Favre I, Kraftsik R, Esteban M, Lobrinus A, Miklossy J (1999). Primary motor cortex involvement in Alzheimer disease. J Neuropathol Exp Neurol.

[CR58] Taira K, Bujo H, Hirayama S, Yamazaki H, Kanaki T, Takahashi K, Ishii I, Miida T, Schneider WJ, Saito Y (2001). LR11, a mosaic LDL receptor family member, mediates the uptake of ApoE-rich lipoproteins in vitro. Arterioscler Thromb Vasc Biol.

[CR59] Verghese PB, Castellano JM, Holtzman DM (2011). Roles of Apolipoprotein E in Alzheimer’s disease and other neurological disorders. Lancet Neurol.

[CR60] Villain N, Fouquet M, Baron JC, Mézenge F, Landeau B, de La Sayette V, Viader F, Eustache F, Desgranges B, Chételat G (2010). Sequential relationships between grey matter and white matter atrophy and brain metabolic abnormalities in early Alzheimer’s disease. Brain.

[CR61] Wang L, Zang Y, He Y, Liang M, Zhang X, Tian L, Wu T, Jiang T, Li K (2006). Changes in hippocampal connectivity in the early stages of Alzheimer’s disease: evidence from resting state fMRI. Neuroimage.

[CR62] Wang J, Wang X, He Y, Yu X, Wang H, He Y (2015). Apolipoprotein E ε4 modulates functional brain connectome in Alzheimer’s disease. Hum Brain Mapp.

[CR63] Wang P, Zhou B, Yao H, Zhan Y, Zhang Z, Cui Y, Xu K, Ma J, Wang L, An N, Zhang X, Liu Y, Jiang T (2015). Aberrant intra- and inter-network connectivity architectures in Alzheimer’s disease and mild cognitive impairment. Sci Rep.

[CR64] Westlye ET, Lundervold A, Rootwelt H, Lundervold AJ, Westlye LT (2011). Increased hippocampal default mode synchronization during rest in middle-aged and elderly APOE epsilon4 carriers: relationships with memory performance. J Neurosci.

[CR65] Winkler AM, Ridgway GR, Webster MA, Smith SM, Nichols TE (2014). Permutation inference for the general linear model. Neuroimage.

[CR66] Wisdom NM, Callahan JL, Hawkins KA (2011). The effects of apolipoprotein E on non-impaired cognitive functioning: a meta-analysis. Neurobiol Aging.

[CR67] Yu C, Zhou Y, Liu Y, Jiang T, Dong H, Zhang Y, Walter M (2011). Functional segregation of the human cingulate cortex is confirmed by functional connectivity based neuroanatomical parcellation. Neuroimage.

[CR68] Zhou Y, Dougherty JH, Hubner KF, Bai B, Cannon RL, Hutson RK (2008). Abnormal connectivity in the posterior cingulate and hippocampus in early Alzheimer’s disease and mild cognitive impairment. Alzheimers Dement.

