# Supplementary material for: Modulation of APOE and SORL1 genes on hippocampal functional connectivity in healthy young adults
Source: Brain Struct Funct. 2017 Feb 22;222(6):2877–89. doi: 10.1007/s00429-017-1377-3 (PMC5541082; doi:10.1007/s00429-017-1377-3)
Supplement: Supplementary file 1 — Supplementary material 1 (DOC 8232 KB) [file 429_2017_1377_MOESM1_ESM.doc]

**Supplementary Materials**

**Supplementary material and methods**

*Genotyping*

The PCR primer sequences of SORL1 rs2070045 were as follows: forward: 5′TCTCACCTGCAGAAATGCAC 3′, reverse 5′GCCTAGATTTTCCGATCGTTT 3′. PCR was performed with a 20μL reaction volume containing 1μL genomic DNA, 0.4μL primer mixture, 2μL dNTPs, 0.6μL Mg^2+^, 2μL buffer, 4μL Q-Solution, and 0.3μL Taq DNA polymerase. The amplification protocol consisted of an initial denaturation and enzyme activation phase at 95°C for 15 min followed by 35 cycles of denaturation at 94°C for 30s, annealing for 1min and 30s at 56°C, extension at 72°C for 1 min, and then a final extension at 72°C for 7 min. PCR products were verified in 3% agarose gels that had been stained with ethidium bromide to regulate the amount of DNA added to the LDR.

Three probes were designed for the LDR reactions, including one common probe (rs2070045, P-GACCAGTCCCTGCAGTCGTTGTCCCTTTTTTTTTTTTTTTTTTTTTTTTTTTTTTTTTT-FAM) and two discriminating probes for the two alleles (rs2070045_G, TTTTTTTTTTTTTTTTTTTTTTTTTTTTTTTTTTCTGACCGGTACAGTTGGCTTCATCC; rs2070045_T, TTTTTTTTTTTTTTTTTTTTTTTTTTTTTTTTTTTTCTGACCGGTACAGTTGGCTTCATCA). These reactions were carried out in a 10μL mixture containing 1μL buffer, 1μL probe mix, 0.05μL Taq DNA ligase, 1μL PCR product, and 6.95μL deionized water. The reaction program consisted of an initial heating at 95°C for 2min followed by 35cycles of 30s at 94°C and 2min at 50°C. Reactions were stopped by chilling the tubes in an ethanol-dry ice bath and adding 0.5mL of 0.5mM EDTA. Aliquots of 1μL of the reaction products were mixed with 1μL of loading buffer (83% formamide, 8.3 mM EDTA and 0.17% blue dextran) and 1μL ABI GS-500 Rox-Fluorescent molecular weight marker, denatured at 95°C for 2min. The samples were the chilled rapidly on ice prior to being loaded on an 5M urea-5% polyacrylamide gel and electrophoresed on an ABI 3100 DNA sequencer at 3000V. Finally, the fluorescent ligation products were analyzed and quantified using the ABI GeneMapper software. APOE genotype for each subject was determined by testing the genotype of two single nucleotide polymorphisms within the gene (rs7412 and rs429358) using PCR amplification followed by direct DNA sequencing.

*Gray matter volume (GMV) analysis*

The VBM analysis was performed using SPM8 ([http://www.fil.ion.ucl.ac.uk/spm/software/spm8](http://www.fil.ion.ucl.ac.uk/spm/software/spm8/)). The structural MR images were segmented into gray matter, white matter and cerebrospinal fluid using the standard unified segmentation model in SPM8. Following segmentation, gray matter population templates were generated from the entire image dataset using the diffeomorphic anatomical registration through the exponentiated Lie algebra (DARTEL) technique (Ashburner, 2007). After an initial affine registration of the gray matter DARTEL template to the tissue probability map in MNI space (http://www.mni.mcgill.ca/), non-linear warping of gray matter images was performed to the DARTEL gray matter template in MNI space with a resolution of 1.5 mm^3^ (as recommended for the DARTEL procedure). The gray matter volume (GMV) of each voxel was obtained by multiplying the gray matter concentration map by the non-linear determinants derived from the spatial normalization step. Finally, to compensate for residual between-subject anatomical differences, the GMV images were smoothed with a full width at half maximum (FWHM) kernel of 8 mm. In effect, here the regional GMV represents normalized GMV after removing the confounding effect of variance in individual brain sizes. After these processing steps, the normalized, modulated, and smoothed GMV maps were used for statistical analysis.

*Comparison between risk-allele carriers*

We extracted hippocampal rsFC values with the PCC, the Pcu, the left MTG and the left IFG from each risk-allele carrier. General linear model was used to compare hippocampal connectivity differences (*P* < 0.05) between double and one risk-allele carriers while controlling for the effects of age, sex and educational years.

**Supplementary references**

Ashburner J (2007) A fast diffeomorphic image registration algorithm. NeuroImage 38:95-113.

**Supplementary figures**


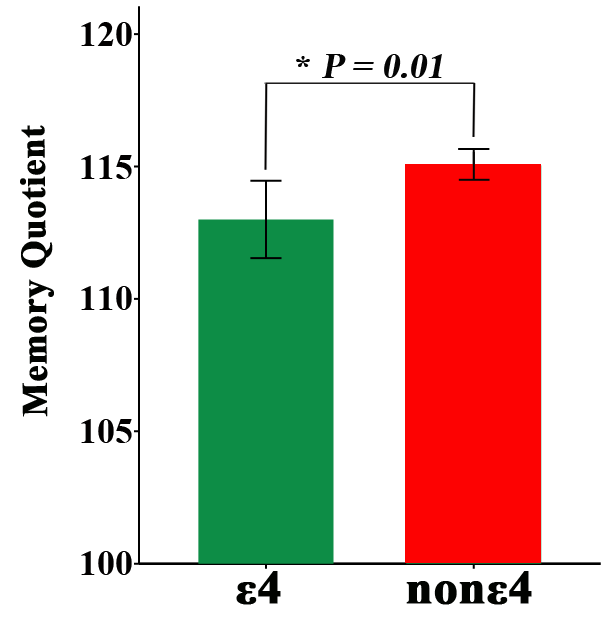


**Figure S1.** Main effect of *APOE* on memory quotient. *APOE* ε4 carriers have significantly reduced memory quotient than APOE non-ε4 carriers.


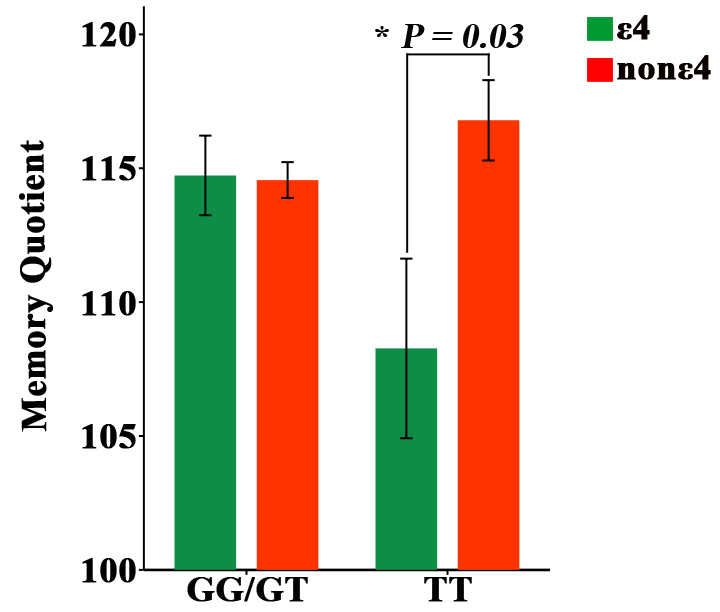


**Figure S2.** Interaction effect of *SORL1* and *APOE* on memory quotient. In *SORL1* TT carriers, subjects with risk *APOE* ε4 allele show significantly reduced memory quotient than non-ε4 carriers.


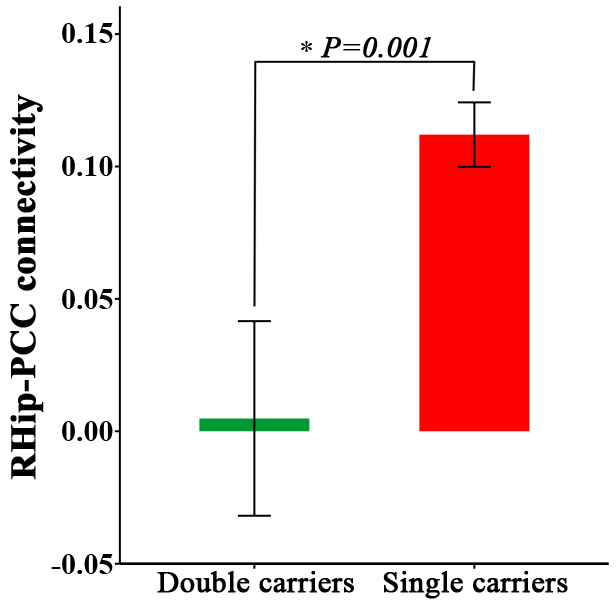


**Figure S3.** The right hippocampal connectivity with the PCC in double (n = 30) and single (n = 204) risk-allele carriers. PCC, posterior cingulate cortex; RHip, right hippocampus.


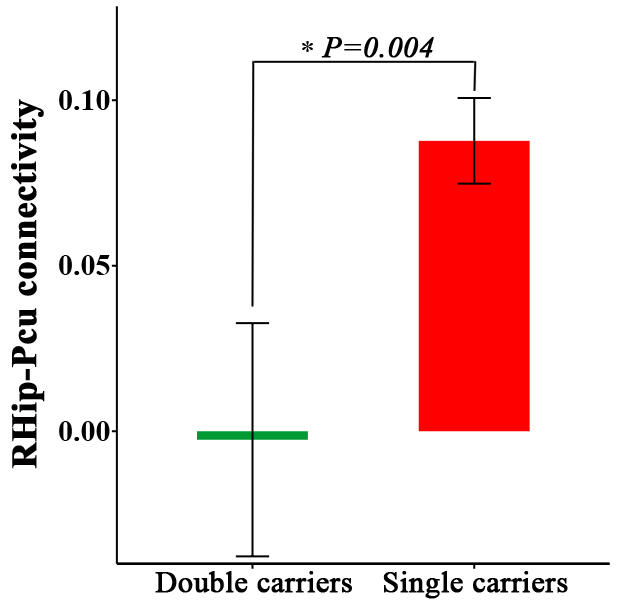


**Figure S4.** The right hippocampal connectivity with Pcu in double (n = 30) and single (n = 204) risk-allele carriers. RHip, right hippocampus; RPcu, right precuneus.

**
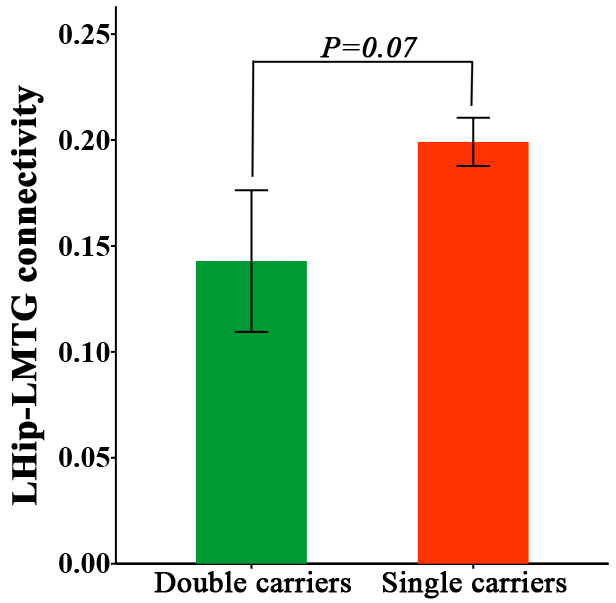
**

**Figure S5.** The left hippocampal connectivity with the left MTG in double (n = 30) and single (n = 204) risk-allele carriers. LHip, left hippocampus; LMTG, left middle temporal gyrus.


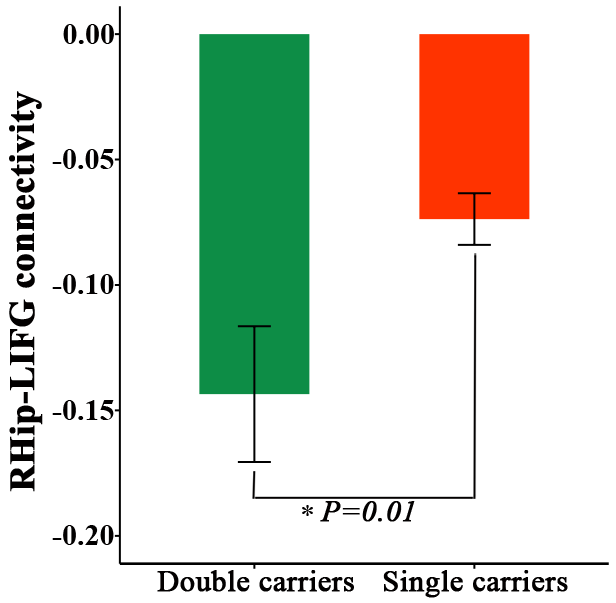


**Figure S6.** The right hippocampal connectivity with the left IFG in double (n = 30) and single (n = 204) risk-allele carriers. LIFG, left inferior frontal gyrus; RHip, right hippocampus.


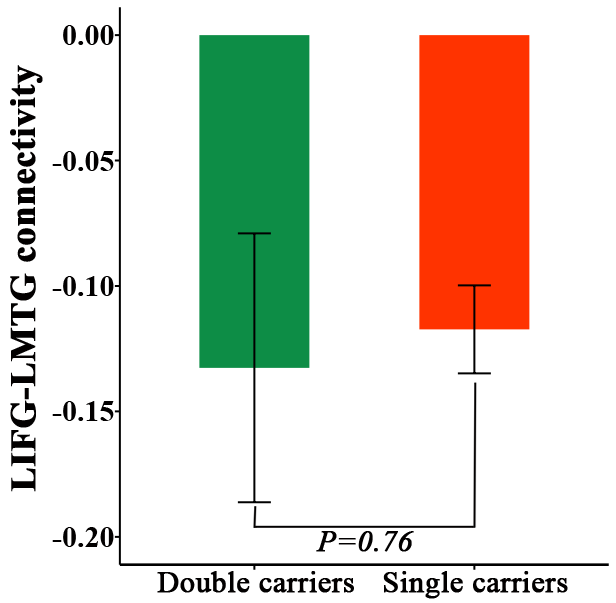


**Figure S7.** The left IFG connectivity with the left MTG in double (n = 30) and single (n = 204) risk-allele carriers. LIFG, left inferior frontal gyrus; LMTG, left middle temporal gyrus.


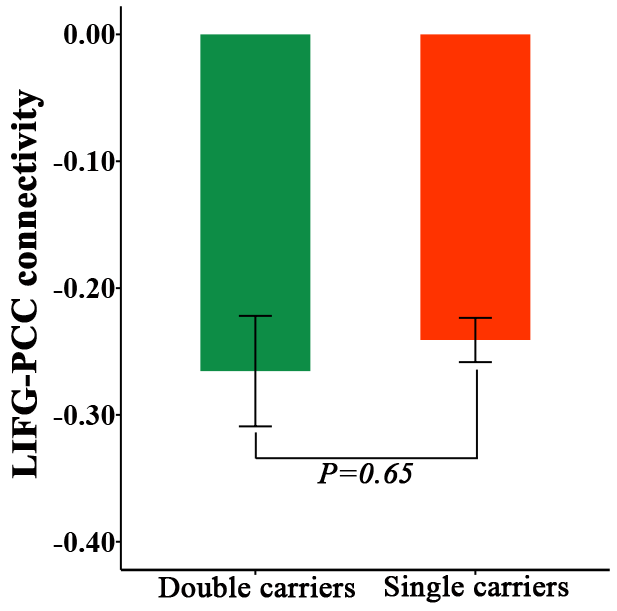


**Figure S8.** The left IFG connectivity with PCC in double (n = 30) and single (n = 204) risk-allele carriers. LIFG, left inferior frontal gyrus; PCC, posterior cingulate cortex.


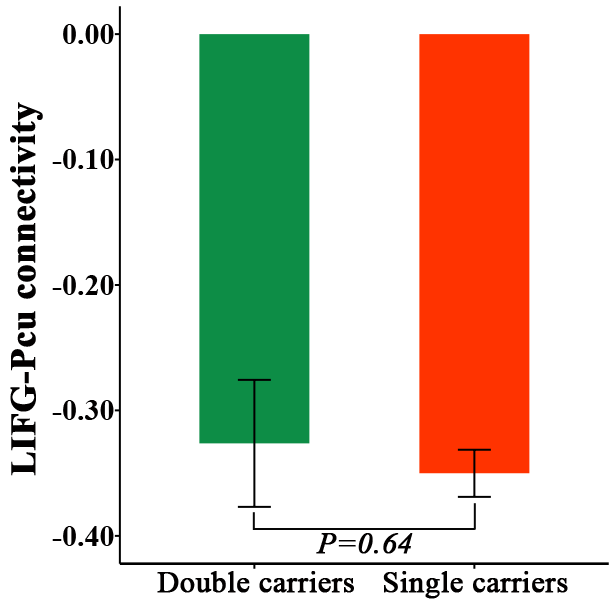


**Figure S9.** The left IFG connectivity with Pcu in double (n = 30) and single risk-allele (n = 204) carriers. LIFG, left inferior frontal gyrus; Pcu, precuneus.
